# PGC-1α overexpression protects against aldosterone-induced podocyte depletion: role of mitochondria

**DOI:** 10.18632/oncotarget.7859

**Published:** 2016-03-02

**Authors:** Min Zhao, Yanggang Yuan, Mi Bai, Guixia Ding, Zhanjun Jia, Songming Huang, Aihua Zhang

**Affiliations:** ^1^ Department of Nephrology, Nanjing Children's Hospital, Nanjing Medical University, Nanjing, China; ^2^ Jiangsu Key Laboratory of Pediatrics, Nanjing Medical University, Nanjing, China; ^3^ Department of Nephrology, The First Affiliated Hospital of Nanjing Medical University, Nanjing, China

**Keywords:** PGC-1α, podocytes, aldosterone, mitochondria, podocyte loss, Pathology Section

## Abstract

Growing evidence has shown that podocyte number is a critical determinant for the development of glomerulosclerosis and progressive renal failure. We previously reported that mitochondrial dysfunction (MtD) is an early event in podocyte injury. Peroxisome proliferator-activated receptor-γ coactivator-1α (PGC-1α) is an important modulator of mitochondrial biogenesis. Here, we investigated the role of PGC-1α overexpression in podocyte depletion and the involvement of mitochondria in this process. Following chronic aldosterone (Aldo) infusion for 14 days, we observed a remarkable podocyte loss, podocyte phenotypic changes, and albuminuria in WT mice. However, all these abnormalities were significantly attenuated in PGC-1α transgenic mice. Next, we examined mitochondrial function in both genotypes with or without Aldo infusion. As expected, Aldo-induced MtD in glomeruli was markedly improved in PGC-1α transgenic mice. In vitro, Aldo induced podocyte detachment and phenotypic changes in line with MtD in dose- and time-dependent manners. Similarly, ethidium bromide, an inducer of MtD, mimicked Aldo effects on podocyte detachment and phenotypic alterations. Notably, overexpression of PGC-1α in podocytes entirely reversed Aldo-induced podocyte detachment, phenotypic changes, and MtD. Taken together, these findings demonstrate that PGC-1α protects against podocyte depletion and phenotypic changes possibly by maintaining normal mitochondrial function.

## INTRODUCTION

Podocytes are highly differentiated glomerular epithelial cells with limited potential to divide. They are primarily responsible for maintaining the integrity of the glomerular basement membrane, and abnormal podocyte morphology and dysfunction are involved in proteinuria [1]. Moreover, podocyte depletion is a major mechanism driving glomerulosclerosis, which leads to end-stage renal disease (ESRD) [2, 3].

Mitochondria are complex intracellular organelles that are responsible for various metabolic functions, including energy production via oxidative phosphorylation. Mitochondria are also a major source of reactive oxygen species (ROS), and the overproduction of ROS damages mitochondrial DNA (mtDNA) and the oxidation respiratory chain, which ultimately causes mitochondrial dysfunction (MtD) [4]. In general, MtD is characterized by increased ROS production, the accumulation of impaired mtDNA, and progressive respiratory chain dysfunction [5-7]. We previously demonstrated that MtD is an early event in aldosterone (Aldo)-induced podocyte injury [8]. However, the role of MtD in podocyte loss remains unknown.

Peroxisome proliferator-activated receptor-γ coactivator-1α (PGC-1α), the first identified cofactor of the nuclear hormone receptor PPAR and other nuclear hormone receptors, potently modulates mitochondrial biogenesis and function [9]. PGC-1α co-activates the transcriptional function of nuclear respiratory factor-1, which specifically binds to the mitochondrial transcription factor A (TFAM) promoter to drive the transcription of TFAM, a direct regulator of mtDNA replication. PGC-1α is also an important regulator of diverse metabolic pathways in response to environmental and physiological changes [10]. The overexpression of PGC-1α increases oxidative capacity in cultured myotubes by improving lipid metabolism and increasing the expression of genes involved in mitochondrial function and biogenesis. The overexpression of PGC-1α also decreases apoptosis in adipose-derived stem cells by reducing intracellular and mitochondrial ROS levels [11]. *In vivo*, specific PGC-1α overexpression in the muscle increases exercise performance and peak oxygen uptake [12]. However, the role of PGC-1α in podocyte loss has not been defined.

Recent clinical and experimental studies have shown that Aldo is a potent contributor of podocyte injury. Inhibition of Aldo action via the antagonism of mineralocorticoid receptor (MR) could attenuate the severity of proteinuria and slow the progression of CKD [13-15]. In the present study, we employed transgenic approaches to fully investigate the role of the PGC-1α/mitochondria axis in modulating Aldo-induced podocyte loss and phenotypic alteration. In detail, we examined the ability of 1) PGC-1α overexpression in mice to protect against podocyte loss and MtD, 2) MtD to confer Aldo-induced podocyte loss, and 3) PGC-1α overexpression in cells to block Aldo-induced MtD and podocyte detachment.

## RESULTS

### PGC-1α overexpression in mice attenuated podocyte depletion and phenotypic changes induced by chronic Aldo infusion

**Figure 1:**
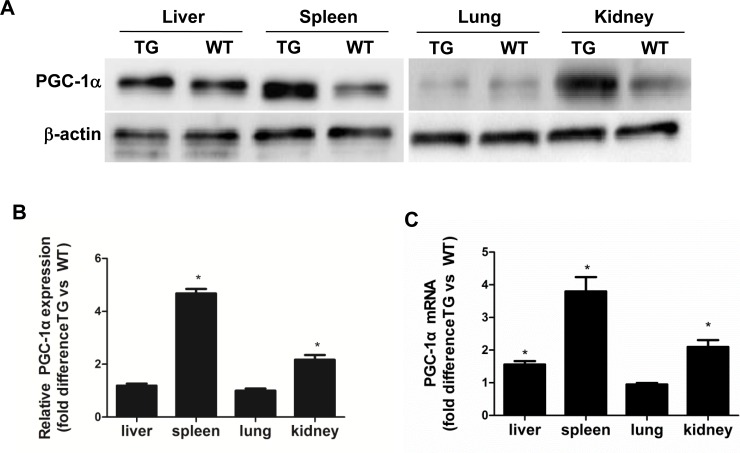
Characterization of PGC-1α transgenic mice **A.** PGC-1α protein levels in the liver, spleen, lung, and kidney. **B.** Densitometric analysis of PGC-1α protein levels. **C.** Ratio of PGC-1α mRNA levels in the kidney cortex and other tissues from TG mice compared with WT controls, as determined by qRT-PCR. The results are presented as means ± SE (*n* = 8). *P < 0.05 *vs*. WT Cntl.

**Figure 2:**
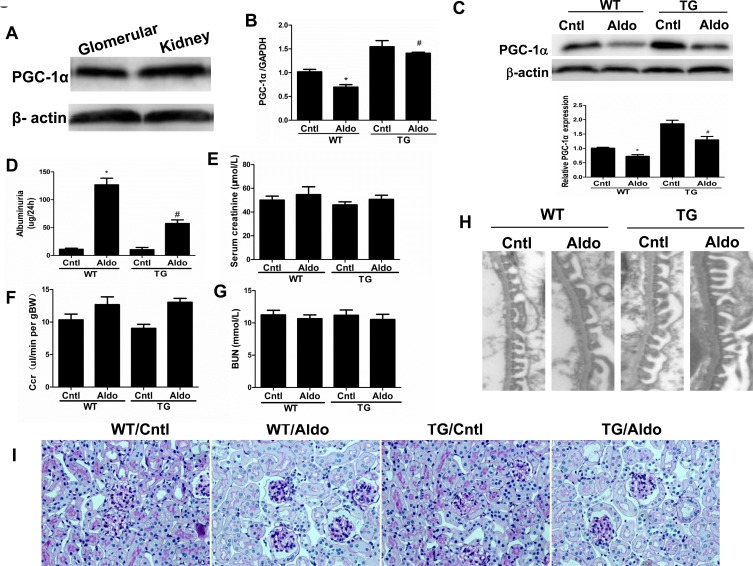
Overexpression of PGC-1α in mice blocked aldosterone-induced podocyte injury Male WT and TG mice were infused with Aldo (300 μg/kg/d) for two weeks. **A.** Western blotting analysis for PGC-1α in glomeruli and kidney. **B.** qRT-PCR analysis of PGC-1α in the kidney cortex. **C.** Western blotting analysis for PGC-1α. Upper: representative immunoblots; Lower: densitometric analysis. **D.** Urinary albumin excretion. **E.** Creatinine (mg/dl). **F.** Endogenous creatinine clearance rate (CCR). **G.** Blood urea nitrogen (BUN). **H.** Foot processes of podocytes by transmission electron microscopy (TEM). **I.** Periodic Acid-Schiff staining. The results are presented as means ± SE (*n* = 8). *P < 0.05 *vs*. control. #*P* < 0.05 *vs*. Aldo+WT.

To investigate the role of PGC-1α in the pathogenesis of podocyte loss, PGC-1α TG mice were subjected to chronic Aldo infusion. First, the expression of PGC-1α in various tissues from WT and TG mice was measured by qRT-PCR and Western blotting. As shown in Figure 1A-1C, PGC-1α expression was significantly increased by ∼2-fold in the kidney and ∼4-fold in the spleen in TG mice as compared with WT controls. In other tissues, PGC-1α expression did not significantly differ between genotypes possibly due to the low activity of the promoter driving this transgene. In glomeruli, PGC-1α protein expression was comparable to the kidney cortex tissues in PGC-1α transgenic mice (Figure 2A).

**Figure 3:**
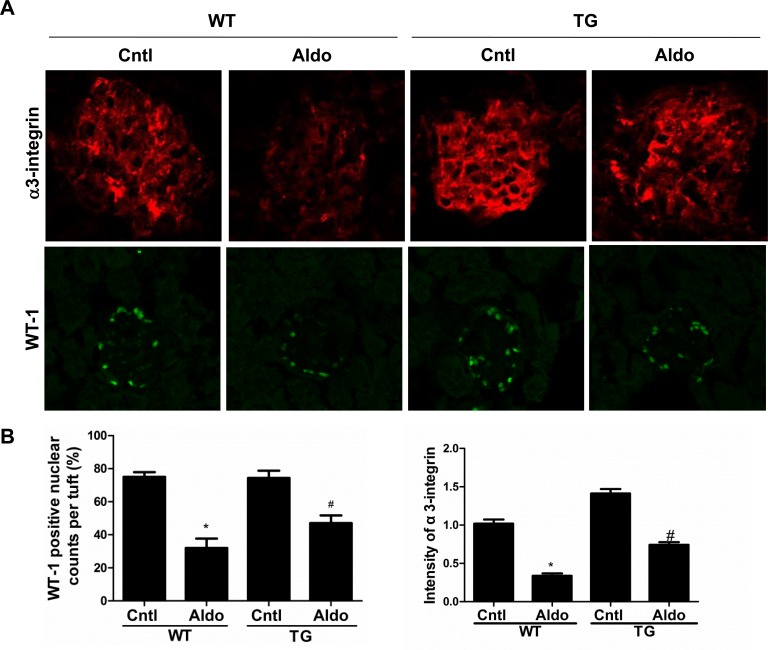
Overexpression of PGC-1α blocked podocyte loss in aldosterone-infused mice **A.** immunofluorescence for α3-integrin and WT1; **B.** quantification of WT1-positive cells and the fluorescence intensity of α3-integrin. The results are presented as means ± SE (*n* = 4). *P < 0.05 *vs*. Cntl. #*P* < 0.05 *vs*. Aldo+WT.

**Figure 4:**
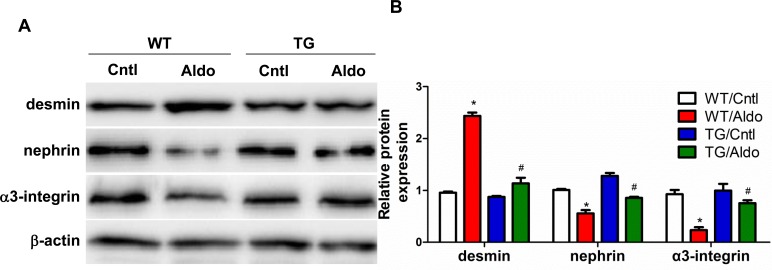
Overexpression of PGC-1α blocked podocyte phenotypic changes in aldosterone-infused mice **A.** Representative immunoblots of desmin, nephrin, and α3-integrin. **B.** densitometric analysis. The results are presented as means ± SE (*n* = 4). *P < 0.05 *vs*. Cntl. #*P* < 0.05 *vs*. Aldo+WT.

Aldo treatment slightly but significantly reduced PGC-1α expression at the mRNA (Figure 2B) and protein (Figure 2C) levels in the kidneys of WT mice. However, kidney PGC-1α expression was significantly higher in TG mice than in WT controls, irrespective of Aldo treatment (Figure 2B and 2C). PGC-1α transgenic mice exhibited improved albuminuria (Figure 2D), while the kidney function evidenced by BUN, blood creatinine and creatinine clearance displayed no difference between genotypes with or without Aldo treatment (Figure 2E-2G). In line with the reduced proteinuria, EM indicated obviously ameliorated podocyte foot process effacement in PGC-1α TG mice following 2 weeks of Aldo infusion (Figure 2H). We then examined the glomerular morphology by PAS staining and found that Aldo-infused WT mice showed slight glomerular enlargement and a larger mesangial area, but these changes were not observed in TG mice (Figure 2I). Immunofluorescence indicated significantly fewer WT-1-positive cells in Aldo-treated WT mice, whereas this reduction was significantly attenuated in TG mice (Figure 3A and 3B), suggesting an improvement in podocyte depletion. Subsequently, we examined α3-integrin expression by Western blotting and immunofluorescence and found that Aldo significantly decreased the expression of α3-integrin (an important protein for maintaining podocyte integrity) in WT mice, and this decrease was remarkably blunted in PGC-1α TG mice (Figure 3A and 3B and Figure 4A and 4B). The pattern of nephrin expression was similar to that of α3-integrin expression (Figure 4A and 4B). In addition, the Aldo-induced reduction in desmin expression was blunted in PGC-1α TG mice (Figure 4A and 4B).

### PGC-1α overexpression in mice attenuated Aldo-induced mitochondrial dysfunction in kidneys

**Figure 5:**
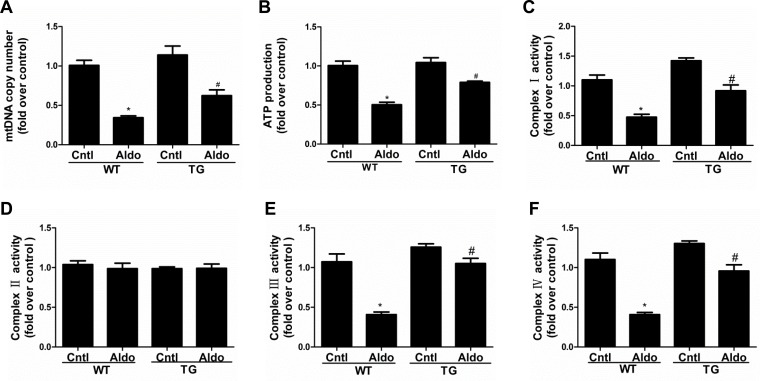
Overexpression of PGC-1α in mice blocked Aldo-induced mitochondrial dysfunction Male WT and TG mice were infused with Aldo (300 μg/kg/d) for two weeks. **A.** mtDNA copy number was analyzed by qRT-PCR. **B.** ATP content was tested with a bioluminescence assay kit. **C.**-**F.** Activities of mitochondrial respiratory chain enzyme complexes I **C.**, II **D.**, III **E.**, and IV **F.**. The results are presented as means ± SE (*n* = 8). *P < 0.05 *vs*. Cntl. #*P* < 0.05 *vs*. Aldo-treated WT mice.

Consistent with our previous study [16], two weeks of Aldo infusion remarkably decreased the mtDNA copy number and ATP content in the kidneys of WT mice (Figure 5A and 5B), and these decreases were markedly blocked in PGC-1α TG mice. As shown in Figure 5C, 5E and 5F, PGC-1α overexpression also restored the activities of complexes I, III, and IV. However, the activity of complex II was not affected by Aldo infusion or PGC-1α overexpression (Figure 5D). Taken together, these data indicate that PGC-1α plays an important role in protecting mitochondria function following long-term Aldo challenge. The beneficial effects of PGC-1α on mitochondria might be, at least in part, attributable to improved podocyte depletion and phenotypic alteration.

### Aldo caused podocyte detachment *in vitro*

**Figure 6:**
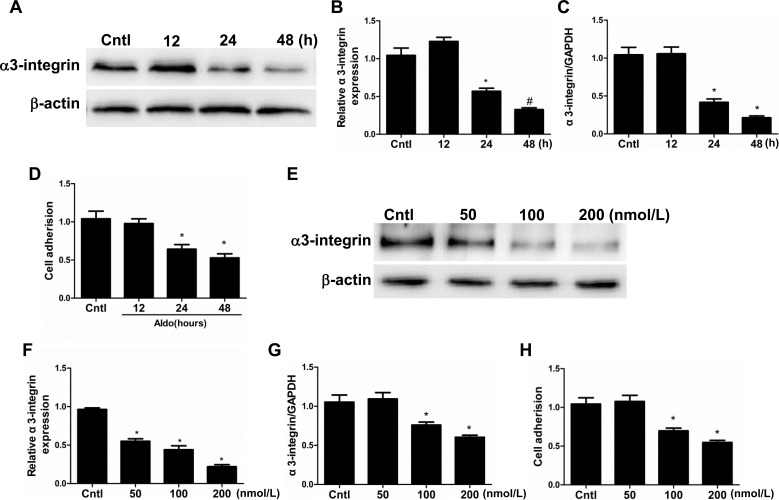
Aldo induced podocyte detachment Cells were grown on 6-well plates until 80% confluence and then treated with Aldo (100 nmol/l) for 12 h, 24 h, and 48 h (A-D) or various doses of Aldo (50, 100, and 200 nmol/l) for 24 h (E-H). **A.** Western blotting analysis of α3-integrin. **B.** densitometric analysis of α3-integrin. **C.** qRT-PCR analysis of α3-integrin. **D.** Cell adhesion was analyzed using an ECM Cell Adhesion Assay. **E.** Western blotting analysis of α3-integrin treated by different dose of Aldo. **F.** densitometric analysis of α3-integrin. **G.** qRT-PCR analysis of α3-integrin. **H.** Cell adhesion was analyzed using an ECM Cell Adhesion Assay. The results are presented as means ± SE (*n* = 4). *P < 0.05 *vs*. Cntl.

The expression of α3-integrin, an adhesion molecule expressed in the podocyte basal membrane domain that maintains podocyte integrity, was examined by Western blotting and qRT-PCT. Specifically, the expression of α3-integrin decreased in a time- and dose-dependent manner at both the protein and mRNA level (Figure 6A-6C and 6E-6G). Similarly, podocyte adhesion (ECM Cell Adhesion Assays) was also significantly decreased in both a time- and dose-dependent manner (Figure 6D and 6H). These data indicate that Aldo directly affects podocyte detachment and downregulates α3-integrin, and the latter may contribute to podocyte depletion.

### Aldo altered podocyte phenotypes *in vitro*

**Figure 7:**
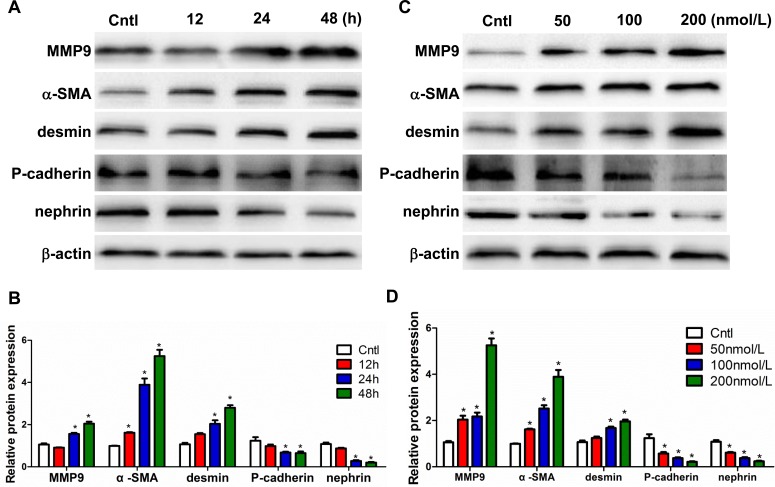
Aldo induced podocyte phenotypic changes in a time- and dose-dependent manner Cells were grown on 6-well plates until 80% confluence and then treated with Aldo (100 nmol/l) for 12 h, 24 h, and 48 h or various doses of Aldo (50, 100, and 200 nmol/l) for 24 h. **A.**, **C.** Representative immunoblots of MMP9, α-SMA, desmin, P-cadherin, and nephrin protein expression by Western blotting. **B.**, **D.** densitometric analysis. The results are presented as means ± SE (*n* = 4). *P < 0.05 *vs*. Cntl.

After treatment with 100 nmol/l Aldo, the protein levels of MMP9, α-SMA, and desmin increased in a time-dependent manner (Figure 7A and 7B). In contrast, Aldo significantly reduced the levels of P-cadherin and nephrin a time-dependent manner (Figure 7A and 7B). Consistently, treatment with various doses of Aldo (0, 50, 100, and 200 nmol/L) affected the expression of all above-described parameters in a dose-dependent manner (Figure 7C and 7D). Taken together, these data demonstrate that Aldo directly affects the phenotypes of podocytes.

### Mitochondrial dysfunction induced podocyte detachment and phenotypic changes

**Figure 8:**
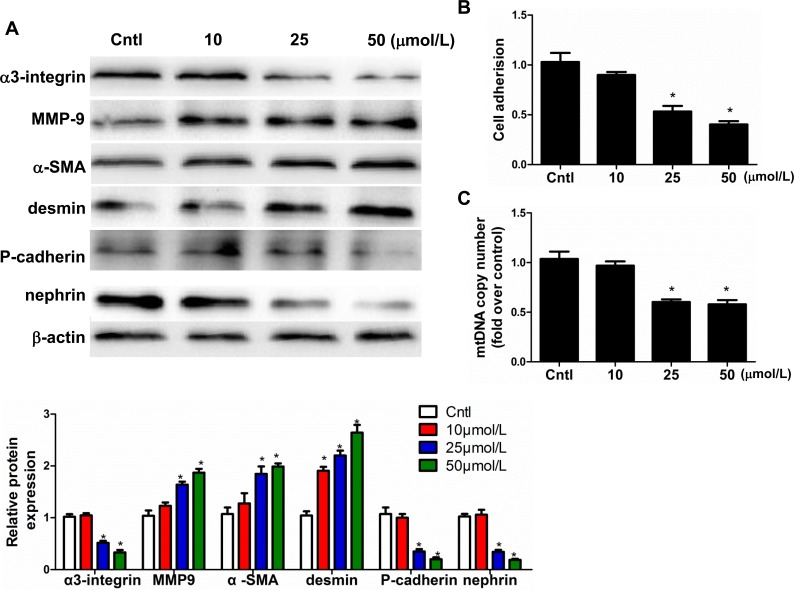
Mitochondrial dysfunction induced podocyte detachment Cells were grown on 6-well plates until 80% confluence and then treated with EtBr (10, 25, and 50 μmol/l) for 24h.**A.** Western blotting analysis for α3-integrin, MMP-9, α-SMA, desmin, P-cadherin, nephrin, Upper: representative immunoblots. Lower: densitometric analysis. **B.** Cell adhesion was analyzed using an ECM Cell Adhesion Assay. **C.** mtDNA copy number. The results are presented as means ± SE (*n* = 4). *P < 0.05 *vs*. Cntl.

Based on our previous work showing that Aldo causes mitochondrial dysfunction in podocytes [16], we investigated the ability of mitochondrial dysfunction to result in podocyte detachment and phenotypic changes. Exposure to low concentrations of EtBr selectively reduces the mtDNA content in numerous cell types, and this effect is reversible after EtBr removal. As shown in Figure 8A and 8B, EtBr strikingly caused podocyte detachment, which corroborates the observed reductions in α3-integrin expression and mitochondrial copy number. Moreover, the expression levels of MMP9, α-SMA, and desmin significantly increased, whereas those of P-cadherin and nephrin significantly decreased in dose-dependent manner (10, 25, and 50 μmol/l). EtBr also affected the podocyte phenotype in a dose-dependent manner (Figure 8A). These findings all suggest that mitochondrial dysfunction plays a crucial role in mediating Aldo-induced podocyte loss and phenotypic changes.

### PGC-1α overexpression in podocytes attenuated Aldo-induced cell detachment and phenotypic alterations in line with improved mitochondrial function

**Figure 9:**
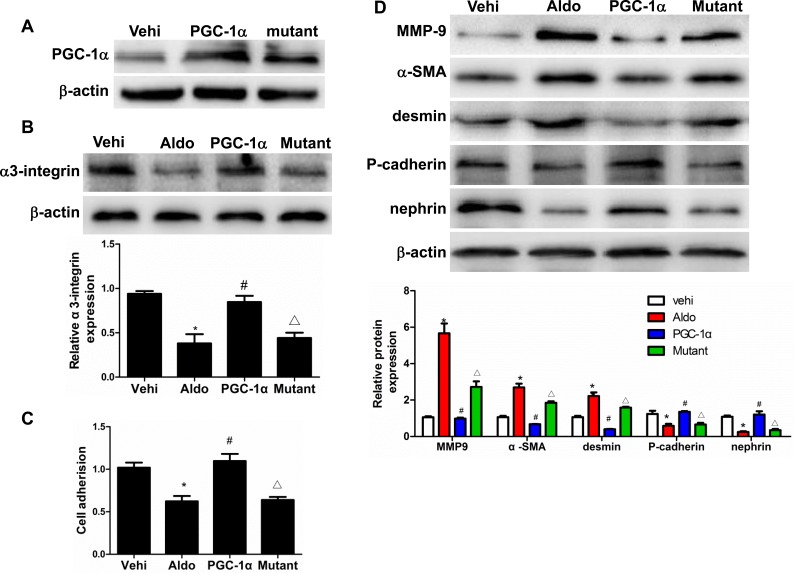
Overexpression of PGC-1α in podocytes blocked Aldo-induced podocyte phenotypic changes Cells were grown on 6-well plates until 30% confluence, transfected with Lentivirus expressing PGC-1α or mutant plasmid for 24 h, and then treated with Aldo (200 μmol/l) for another 24 h. **A.** Western blotting analysis of PGC-1α. **B.** Western blotting analysis for α3-integrin, Upper: representative immunoblots. Lower: densitometric analysis. **C.** Cell adhesion analysis. **D.** Western blotting analysis for MMP-9, α-SMA, desmin, P-cadherin, nephrin, Upper: representative immunoblots. Lower: densitometric analysis. The results are presented as means ± SE (*n* = 4). *P < 0.05 *vs*. vehi group. #*P* < 0.01 *vs*. Aldo group. Δ*P* < 0.05 *vs*. PGC-1α overexpression group.

**Figure 10:**
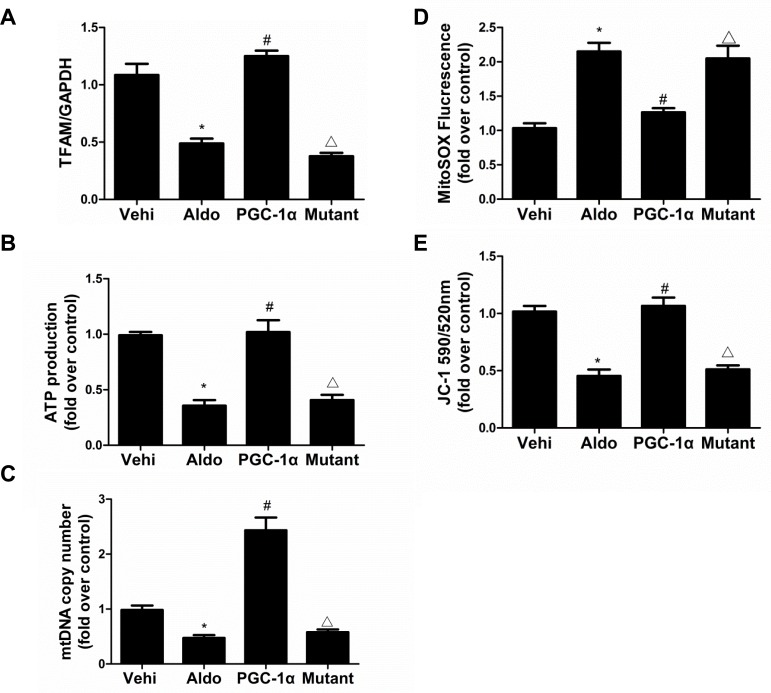
Overexpression of PGC-1α in podocytes blocked Aldo-induced mitochondrial dysfunction Cells were grown on 6-well plates until 30% confluence, transfected with lentivirus expressing PGC-1a and mutant plasmid for 24 h, and then treated with Aldo (200 μmol/l) for another 24 h. **A.** qRT-PCR analysis of TFAM, **B.** adenosine 5′-triphosphate (ATP), and **C.** mitochondrial DNA (mtDNA) copy number. **D.** Mitochondrial ROS levels. **E.** Mitochondrial membrane potential content. The results are presented as means ± SE (*n* = 4). *P < 0.05 *vs*. Cntl. #*P* < 0.01 *vs*. Aldo group. #*P* < 0.05 *vs*. PGC-1α overexpression group.

To further examine the direct role of PGC-1α in Aldo-induced podocyte depletion, we overexpressed PGC-1α in podocytes. As shown in Figure 9A-9C, the overexpression of PGC-1α entirely reversed the Aldo-induced reduction in α3-integrin expression and podocyte detachment. Moreover, PGC-1α overexpression also attenuated Aldo-induced changes in MMP-9, α-SMA, desmin, P-cadherin, and nephrin expression (Figure 9D), indicating that the podocyte phenotype was maintained. In addition, the overexpression of PGC-1α also significantly protected mitochondrial function, as evidenced by attenuated decreases in TFAM expression, ATP production, mtDNA copy number, and MMP expression and increased ROS production following Aldo treatment (Figure 10A-10E). These data suggest that PGC-1α overexpression in podocytes attenuated Aldo-induced cell detachment and phenotypic changes, possibly by improving mitochondrial function.

## DISCUSSION

Podocyte depletion occurs in many glomerular diseases, including focal segmental glomerulosclerosis (FSGS), diabetic nephropathy, immunoglobulin A nephropathy and lupus nephritis [18]. Recent studies demonstrated that urinary podocyte loss might be a more specific marker of ongoing glomerular damage than proteinuria [19]. The progression of diabetic nephropathy in both type 1 and type 2 diabetes was associated with podocyte loss [20, 21]. Fukuda et al. also reported that persistent podocyte loss induced by angiotensin-II could cause glomerulosclerosis and ESRD [22]. Most recently, Daehn et al. further confirmed the podocyte loss phenomenon in FSGS patients [23]. These findings all strongly suggest that podocyte loss is not only a phenomenon observed in animal models and patients but also a pathogenic factor leading to the progression of kidney diseases. Thus, the prevention of podocyte loss could be a promising strategy for the treatment glomerular diseases.

Aldo, a key regulator of blood pressure and electrolytic balance, has attracted considerable attention for its involvement in the development and progression of chronic kidney disease [14, 17]. We previously demonstrated that the Aldo-induced fibrotic response is mediated by mitochondrial dysfunction in renal proximal tubular cells [24]. Specifically, the overproduction of mitochondria-originated ROS plays a crucial role in this process. In podocytes, Aldo has been shown to induce podocyte apoptosis [25]. However, its role in inducing podocyte loss has not been reported. In the present study, we observed a potent action of Aldo in inducing podocyte depletion *in vivo* and detachment *in vitro*, suggesting a novel mechanism of Aldo in contributing to podocyte damage.

PGC-1 co-activators are important regulators of mitochondrial biogenesis and antioxidant defense. Specifically, PGC-1α function is responsible for MtD in several diseases, such as Alzheimer's disease, Parkinson's disease, and cardiovascular diseases [11, 20, 25, 26]. Dysfunctions in mitochondrial energy metabolism lead to reduced ATP production, impaired calcium buffering, and the generation of reactive oxygen species (ROS) [20]. In mitochondrial biogenesis, PGC-1α plays a central role in the detoxification of ROS by regulating the expression of numerous ROS-detoxifying enzymes [21]. Consistent with these findings, our study demonstrated that the overexpression of PGC-1α in mice remarkably ameliorated Aldo-induced mitochondrial dysfunction, as demonstrated by attenuated reductions in the mtDNA copy number and ATP content in kidneys. Meantime, the podocyte depletion, podocyte phenotypic alteration, and podocyte injury-associated albuminuria were all ameliorated. These data highly suggested an *in vivo* role of PGC-1α in antagonizing podocyte loss and MtD. In agreement with *in vivo* results, *in vitro* studies in podocytes further confirmed a protective effect of PGC-1α overexpression in opposing Aldo-induced podocyte detachment and MtD. However, a limitation of this whole body PGC-1α transgenic mouse model is that we could not rule out the contribution of PGC-1α from other cell types (endothelial cells, inflammatory cells, and so on) in protecting podocytes and attenuating albuminuria.

In summary, we first examined podocyte loss in PGC-1α transgenic mice challenged with excessive Aldo. Consistent findings from both *in vivo* and *in vitro* studies strongly indicated that PGC-1α helps to prevent podocyte loss, possibly by at least in part protecting mitochondrial function. Based on the importance of podocyte depletion and phenotype changes in the development and progression of chronic kidney disease, these novel findings substantially increased our understanding of the pathogenesis of podocyte injury and CKD. Targeting PGC-1α and/or the mitochondria may represent a new therapeutic strategy for the treatment of podocyte loss-related glomerular diseases.

## MATERIALS AND METHODS

### Antibodies and kits

Antibodies against PGC-1α and horseradish peroxidase-conjugated secondary antibodies were purchased from Santa Cruz Biotechnology (Santa Cruz, CA). An anti-β-actin antibody was obtained from Cell Signaling Technology (Beverly, MA). Antibodies against nephrin, MMP9, α-SMA, P-cadherin, and desmin were purchased from Abcam (Cambridge, MA). Antibodies against WT-1 (C-19) and integrin-α3 (C-18) were purchased from Santa Cruz Biotechnology (Santa Cruz, CA). Fluorescently conjugated secondary antibodies were purchased from Cell Signaling Technology (Beverly, MA). The Cell Adhesion kit (Cat. No. CBA-061) was purchased from Cell Biolabs (San Diego, CA).

### Cell culture and lentivirus gene transfer

MPC5 conditionally immortalized mouse podocyte cell line (provided by Peter Mundel of the Mount Sinai School of Medicine and Dr. Jie Ding of Peking University) were cultured and induced to differentiate as described previously [16]. The cells were maintained in RPMI 1640 medium (HyClone, USA) containing 10% heat-inactivated fetal calf serum (Gibco, USA), 100 U/ml penicillin G, and 100 mg/ml streptomycin in a 5% CO_2_ atmosphere. To sustain podocyte proliferation, 10 U/ml of recombinant murine interferon-γ (Sigma, USA) was added to the medium, and the cells were maintained at 33°C. Podocytes were maintained without interferon-γ at 37°C for 10-14 days to induce differentiation before the experiments. Lentivirus expressing PGC-1α and mutant were from Santa Cruz Biotechnology. Cells were infected with lentiviruses for 24h before the experiments as described previously [16].

### Identification of PGC-1α transgenic mice

The animal study protocols were reviewed and approved by the Institutional Animal Care and Use Committee at Nanjing Medical University, China. A 2.4-kb fragment of complementary DNA (cDNA) was amplified from a Ppargc1a-targeting vector purchased from Addgene (https://www.addgene.org/1026/). After confirming the DNA sequence, the Ppargc1a cDNA was inserted into a cloning vector, pCAG-GFP [28], followed by excision using NheI and XhoI. For microinjection, the 8148-bp fragment was isolated via digestion with Sal I and then purified after gel electrophoresis using the Ultra-Sep Gel Extraction kit (OMEGA, USA). The purified fragment was quantified and diluted to a concentration of 50 μg/ml in injection buffer consisting of 10 mM Tris at pH 7.4 and 0.2 mM ethylenediaminetetraacetic acid (EDTA). Linearized constructs were microinjected into male pronuclei of C57BL/6 mouse fertilized eggs, and the resulting one-cell embryos were placed into the oviducts of pseudo-pregnant females. The founder PGC-1α transgenic mouse was generated on a C57BL6-DBA mixed background. The mice used in this study were backcrossed six times to a C57BL6 genetic background. The genotype of the TG founders was determined using PCR to amplify the PGC-1α BAC DNA, and the expression of the PGC-1α transgene was analyzed by quantitative real-time PCR. The following primers were used to amplify cDNA and genotype transgenic mice: PGC-1α-forward, 5′-ATCTGACCACAAACGATGACCCT-3′; PGC-1α-reverse, 5′-GAGGCATCTTTGAAGTCTAGTTGTCTA-3′. The amplification profile involved an initial denaturation step of 5 min at 94°C and 35 cycles of 30 s at 94°C, 30 s at 55°C and 2 min at 72°C. The amplified products were analyzed by electrophoresis on a 1.5% agarose gel and visualized using ethidium bromide.

### Aldosterone infusion in mice

In brief, 10-week-old PGC-1α transgenic (TG) and wild-type (WT) male mice weighing 25-30 g were treated with Aldo using osmotic subcutaneously implanted mini-pumps. The pumps were placed by making an incision in the right flank region under light 3% isoflurane anesthesia, and they delivered a continuous infusion of Aldo (300 μg/kg/d) (Alzet, Durect, Cupertino, CA) for 14 days. All mice were maintained on a 12-h light-dark cycle in a temperature-controlled (19-21°C) room. They were fed standard rodent chow and had free access to drinking water.

### Isolation of glomeruli and glomerular mitochondria

Glomeruli were isolated using a previously described method [4, 16]. Briefly, mice were anesthetized and the kidney was removed and perfused with 5ml of phosphate-buffered saline, then kidneys were minced into small pieces, digested by collagenase and DNase, and filtered. After washing for 3 times, the glomeruli were collected using a magnet and the purity of glomeruli was confirmed to be about 95% by phase-contrast microscopy. Mitochondria from glomeruli were isolated using a kit purchased from Sigma (MITO-ISO1, Sigma Chemical, St Louis, MO) according to the manufacturer's protocol. The ATP content (Cat# FLAA, Sigma Chemical, St Louis, MO) and the activity of mitochondrial respiratory chain enzyme complexes were detected using commercial kits: complex I Assay Kit (Cat# MS141, Mitosciences, Eugene, OR), Complex II Assay Kit (Cat# MS241, Mitosciences, Eugene, OR), Complex III Assay Kit (Cat# GMS50009, Genmed Scientifics, Shanghai, China), and Complex IV Assay Kit (Cat# MS444, Mitosciences, Eugene, OR).

### Assessment of urinary albumin and renal function

Urinary albumin was measured by a mouse Albumin ELISA kit according to the manufacturer's instructions (Albuwell M Exocell, Philadelphia, USA). Serum creatinine and blood urea nitrogen (BUN) levels were measured using commercial kits following the manufacturers (Bioassay Systems, Hayward, CA) instructions.

### Quantitative real-time PCR (qRT-PCR)

Total RNA from cultured podocytes and isolated glomeruli was extracted using TRIzol reagent (Invitrogen, Carlsbad, CA). Oligonucleotides were designed using Primer3 software (available at http://frodo.wi.mit.edu/) and synthesized by Invitrogen. qRT-PCR was used to detect the mtDNA copy number and the target gene expression. Reverse transcription was performed by using a Transcriptor First Stand cDNA Synthesis kit (Roche, Germany) according to the manufacturer's protocol. Real-time PCR amplification was performed using an ABI 7500 Real-time PCR Detection System (Foster City, CA) with FastStart Universal SYBR Green master mix (Roche, Germany). The cycling conditions were 95°C for 10 min followed by 40 cycles of 95°C for 15 s and 60°C for 1 min. The relative mtDNA copy numbers were normalized to the 18S ribosomal RNA levels encoded by the nuclear DNA, and C_T_ values were used to analyze the mRNA levels based on a standard curve using SDS 2.2.2 software (Applied Biosystems).

### Western blotting

Podocytes or isolated glomeruli were lysed in protein lysis buffer, and the protein concentration was measured as previously reported [29]. Immunoblotting was performed with primary antibodies against PGC-1α (1:200), nephrin (1:200), MMP9 (1:500), α-SMA (1:1000), P-cadherin (1:1000), desmin (1:500), and α-actin (1:1000). The blots were visualized using the Amersham ECL Detection System (Amersham, Buckinghamshire, UK), and a densitometric analysis was performed using Quantity One Software (Bio-Rad).

### Cell adhesion assay

The podocytes were cultured to a density of 5 × 10^5^ cells per microplate well. Subsequently, 5 μl of calcein AM stock solution (Component A) was added to 1 ml of cell suspension, and the mixture was incubated at 37°C for 30 min. The cells were then resuspended in RPMI to a concentration of 5 × 10^6^ cells/ml, and 100 μl of the calcein-labeled cell suspension was then added. The mixture was incubated at 37°C for 1 h and analyzed by flow cytometry according to the manufacturer's instructions (Cell Adhesion Assay Kit, V-13181).

### Immunoreactivity and histological analysis

For the histological analysis, harvested kidneys from mice were fixed overnight in 4% paraformaldehyde (PFA) at 4°C, embedded in paraffin, and sectioned transversely. The kidneys used for the immunofluorescence analysis were fixed for 15 min in 4% PFA on ice, rinsed once in 1× PBS for 10 min and embedded in Tissue-Tek^®^ O.C.T. compound (Sakura Inetek Europe BV). Cryosections were cut to a thickness of 8 μm, and immunostaining was carried out as described previously [29]. Fluorescent images were produced on a Zeiss Axioplan2 imaging microscope. Kidney sections (3 μm) were stained with periodic acid-Schiff (PAS).

### Transmission electron microscopy (TEM)

Kidney cortexes were cut into 1 mm x 1 mm x 1 mm pieces and then post-fixed in 5% glutaraldehyde. Ultrathin sections (60 nm) were cut on a microtome, placed on copper grids, stained with uranyl acetate and lead citrate, and examined under an electron microscope (JEOL JEM-1010, Tokyo, Japanese).

### Statistical analysis

All results are presented as the means ± SE. Data were compared between two groups using a one-way analysis of variance, whereas multiple groups were compared using the Kruskal-Wallis test performed with SPSS 13.0 statistical software (SPSS, Chicago, IL). A value of *P* < 0.05 was indicated statistical significance.
